# Bats Track and Exploit Changes in Insect Pest Populations

**DOI:** 10.1371/journal.pone.0043839

**Published:** 2012-08-31

**Authors:** Gary F. McCracken, John K. Westbrook, Veronica A. Brown, Melanie Eldridge, Paula Federico, Thomas H. Kunz

**Affiliations:** 1 Department of Ecology and Evolutionary Biology, University of Tennessee, Knoxville, Tennessee, United States of America; 2 Areawide Pest Management Research Unit, USDA-ARS, College Station, Texas, United States of America; 3 Department of Microbiology, University of Tennessee, Knoxville, Tennessee, United States of America; 4 Department of Mathematics, Computer Science, and Physics, Capital University, Columbus, Ohio, United States of America; 5 Centre for Ecology and Conservation Biology, Department of Biology, Boston University, Boston, Massachusetts, United States of America; University of Western Ontario, Canada

## Abstract

The role of bats or any generalist predator in suppressing prey populations depends on the predator's ability to track and exploit available prey. Using a qPCR fecal DNA assay, we document significant association between numbers of Brazilian free-tailed bats (*Tadarida brasiliensis*) consuming corn earworm (CEW) moths (*Helicoverpa zea*) and seasonal fluctuations in CEW populations. This result is consistent with earlier research linking the bats' diet to patterns of migration, abundance, and crop infestation by important insect pests. Here we confirm opportunistic feeding on one of the world's most destructive insects and support model estimates of the bats' ecosystem services. Regression analysis of CEW consumption versus the moth's abundance at four insect trapping sites further indicates that bats track local abundance of CEW within the regional landscape. Estimates of CEW gene copies in the feces of bats are not associated with seasonal or local patterns of CEW abundance, and results of captive feeding experiments indicate that our qPCR assay does not provide a direct measure of numbers or biomass of prey consumed. Our results support growing evidence for the role of generalist predators, and bats specifically, as agents for biological control and speak to the value of conserving indigenous generalist predators.

## Introduction

Reductions in biotic diversity and disruption of natural predator-prey relationships in agricultural landscapes promote episodic irruptions of highly destructive insect pests [1]. Many natural enemies of these pests have been removed by agricultural practices, and pest suppression relies on the use of pesticides, planting of genetically modified crops, and practices to disrupt pest life cycles such as tilling and crop rotation. Favored agents for biological control, typically specialized insect predators with high kill potential, are often ineffective, particularly in landscapes dominated by annual crops, because specialist predators cannot survive periods between pest irruptions when their prey are rare [1], [2], [3]. However, generalist predators can be effective for biological control in these systems if, as opportunistic feeders, they can sustain on alternative prey when pest numbers are low, and recruit rapidly to exploit local resurgences in pest numbers [1], [2], [3]. Given these traits of temporal persistence, rapid exploitation, and opportunistic feeding, generalist predators can assume additional and unique roles within contemporary agricultural practices. These roles include suppression of pest numbers below economic thresholds, thus reducing the need for pesticide applications [4], [5], and “resistance breaking”, or delaying the evolution of resistance to pesticides and transgenic crops [1], [6].

Most bats are highly mobile predators of night-flying insects, many of which are significant pests in natural and agricultural ecosystems. Many insectivorous bats are generalist predators [7], [8], [9], and bats often are cited as important agents for the suppression of agricultural pests [10], [11]. However, information linking bats to impacts on pest populations has been limited, in part because conventional techniques for assessing the diets of bats have relied on identifying insect fragments in feces and typically have not allowed taxonomic identification of prey to the species level [12], [13]. This is particularly a problem with soft-bodied insects such as moths, many of which are important agricultural pests. The use of polymerase chain reaction (PCR) and sequence-based assays of prey remains in feces [14] can alleviate this problem and provide the needed taxonomic precision to better document diets of predators. The recent application of DNA barcoding and sequence-based assays confirm the diverse diets of several species of bats [9], [15], [16]. These studies also demonstrate consumption by bats of several economically important moth species [9] and document seasonal variation and the impact of local habitats on what bats eat [17]. Here, we present the first study using a molecular assay to document consumption of a targeted pest species, while simultaneously measuring the pest's abundance in the habitats where the bats feed.

Because of their adverse economic impact on crops, the spatial and temporal dynamics of populations of corn earworm (CEW) moths (*Helicoverpa zea*; Noctuidae, also, known as cotton bollworm) are well characterized. Early in summer, several billion adult CEW exploit seasonally available southerly winds to disperse from the Lower Rio Grande Valley of northeastern Mexico and southern Texas into the Winter Garden area of south-central Texas where they infest silking corn [18], [19], [20]. Within three to four weeks, the next generation of moths emerges and infests cotton and other crops in the region, or engages in additional long distance northward dispersal. Because of cotton's value, insect populations in cotton are suppressed by pesticides and use of *Bacillus thuringiensis* (*Bt*) transgenic cotton. Typically, CEW moth populations remain low in the region from mid- to late-summer, but increase abruptly in late September and early October with the southward migration of insects on winds associated with autumnal cold fronts [21]. These strong seasonal patterns of CEW moth abundances show annual, regional, and local variation depending on weather and agricultural practices such as crop rotations, planting dates, irrigation, and insecticide applications.

Feeding by Brazilian free-tailed bats (*Tadarida brasiliensis*) in the midst of the migrating moth populations has been documented [22]. Research on the diets of these bats based on conventional analysis of insect fragments in feces also links striking increases in moth consumption with the early- and mid-summer migrations and crop infestations described above [7], [8], [13]. Modeling efforts based on economic values of cotton production, the estimated numbers of bats in the region, and assumptions regarding the numbers of CEW in their diet, indicate a mid-value estimate of $1,700,000 for the annual ecosystem services provided by these bats to Winter Garden cotton growers in avoided crop damage and reduced need for pesticides [5]. Modeling efforts also demonstrate that the ecosystem services of the bats persist with the planting of transgenic (*Bt*) varieties of cotton [6].

In this study, we employ an insect species-specific gene marker and quantitative polymerase chain reaction (qPCR) analysis of fecal DNA to investigate the ability of Brazilian free-tailed bats to track and exploit populations of CEW moths in the Winter Garden area. Moth consumption by the bats is documented in relation to independently obtained measures of moth abundances. Our results suggest that Brazilian free-tailed bats in Texas fulfill the requirements of temporal persistence, rapid exploitation, and opportunistic feeding (1,2,3) to function as effective agents for biological pest control in a contemporary production-based agroecosystem.

## Methods

This research was approved under Texas Parks and Wildlife, Scientific Permit Number SPR-0305-058. The University of Tennessee, Knoxville, Institutional Animal Care and Use Committee approved this study under IACUC protocol #780. Collection of insects on private property was approved by land owners.

### (a) qPCR marker development and validation

Night-flying insects of the size consumed by Brazilian free-tailed bats [7], [8] were collected in the Winter Garden region using pheromone and blacklight traps (BioQuip, Model 2851L:22W). Genomic DNA was obtained from 52 adult CEW, a minimum of 28 adults of each of three other common noctuid moth pests in the region (tobacco budworms (*Heliothis virescens*), fall armyworms (*Spodoptera frugiperda*), and beet armyworms (*S. exigua*)), and from one to six specimens of 65 additional insect taxa captured at field sites in light traps (Information S1, Appendix S1). The insects were frozen upon capture and stored at −20°C. DNA was extracted using a Qiagen DNeasy Tissue DNA Extraction kit with minor modifications that included incubation of excised insect abdomens or the entire body of smaller insects overnight in buffer ATL and proteinase K, followed by centrifugation and collection of supernatant to a new tube, then continuation of the manufacturer's protocol. DNA was also extracted from mealworm (*Tenebrio molitor*; Coleoptera) and waxworm (*Galleria mellonella*; Lepidoptera) larvae that were used in controlled feeding experiments employing big brown bats (*Eptesicus fuscus*) (Information S1), and from wing membrane biopsies from four Brazilian free-tailed bats ([23], Information S1). An ∼750 bp portion of the mitochondrial cytochrome oxidase II (COII) gene was amplified from the insect DNA using the conserved primers A-tLEU and B-tLYS [24]. PCRs were carried out in 12 µL volumes, each containing 10 ng DNA, 1× PCR buffer (Promega), 1.25 mM MgCl_2_, 0.10 mM dNTP's, 10 pmol of each primer (Integrated DNA Technologies), 10 ng BSA (Promega), and 1 unit of *Taq* DNA polymerase (Promega). The PCR amplification profile consisted of initial denaturation for 2 min at 95°C, followed by 30 cycles at 95°C for 1 min, 53.5°C for 1 min, and 72°C for 1 min, with a final extension at 72°C for 5 min. PCR products were purified using a MinElute Gel Extraction Kit (Qiagen) and sequenced using the BigDye v3.1 Terminator Cycle Sequencing Kit (Applied Biosystems) using the A-tLEU primer on an ABI 3100 automated sequencer (Applied Biosystems). COII sequences from CEW and the other insects (Genbank Accession numbers HQ677771–HQ677825) were aligned using Sequencher v4.5 (Gene Codes Corporation), and primers that are specific to CEW (Information S1) were designed using Primer3 software [25] to amplify a shorter, 158 bp, internal portion of the COII sequence (moth COII I5Hz-F (5′-TATAATCCCTTCTAATGAAATAAATTCTAA- 3′) and moth COII I5Hz-R (5′-CATCTACTTTTACCCCTAATGATGG- 3′)). Moth primer COII I5Hz-a (5′ FAMd-AGTTCAAGAGTGGATTACATCTGTTGC-BHQ-1 3′) was designed for use as a probe for qPCR.

A standard curve for quantifying the amount of moth DNA present in samples was constructed using a ten-fold dilution series of 2 to 200,000 copies per uL^−1^ of the cloned CEW COII gene [26]. The entire COII region from one CEW moth was cloned using TOPO TA cloning kit (Invitrogen). Plasmids were harvested with FastPlasmid mini kits (Eppendorf).

### (b) Fecal collections

**Figure 1:**
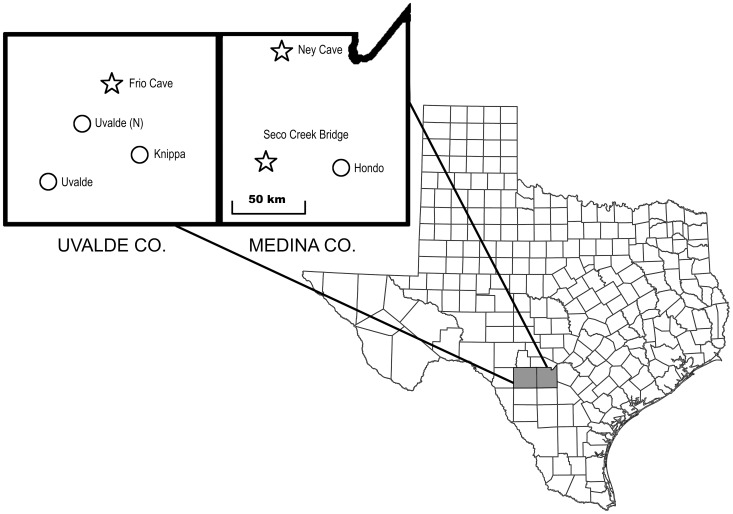
Bat roost sites and insect sampling locations in Texas, U.S. All fecal samples were from bats at the Seco Creek Bridge bat roost (29°19′33.96″N; 99°17′36.53″W). Adult male corn earworm moths were captured in pheromone traps at Hondo (29°17′38.58″N; 99°2′29.94″W), Knippa (29°19′27.84″N; 99°37′0.62″W), Uvalde (N) (29°20′1.61″N; 99°42′46.33″W), and Uvalde (29°15′6.62″N; 99°45′12.78″W) in Medina and Uvalde Counties, Texas. The locations of Ney Cave (29°36′0.54″N; 99°7′39.29″W) and Frio Cave (29°26′5.57″N; 99°41′4.63″W), natural roost sites containing large numbers of Brazilian free-tailed bats, also are shown.

Fecal samples were obtained from free-ranging Brazilian free-tailed bats as they returned at dawn from foraging to a roost under Seco Creek Bridge, Medina County, Texas. Seco Creek Bridge is an Interstate-grade highway bridge located in the northeastern portion of the Winter Garden Region (Figure 1) that houses a seasonal colony of an estimated 100,000 bats [27]. Returning bats were captured using padded hoop nets, a 2 m×1 ½ m harp trap, or a 3-m long mist net, and placed individually within a few minutes of capture in clean cloth bags. After 4 to 6 hours, the bats were removed from bags, feces were collected, and the bats were released at the site of capture. Feces were collected from a total of 634 bats between April 22 and September 18, 2006, typically from 25 bats per morning on two consecutive mornings per week but with a few gaps in the timeline. The fecal sample from each bat was placed in a 2 ml screw cap cryotube (Sarstedt) containing silica gel desiccant (4–10 mesh, Fisher Scientific) and frozen within 4 hours of collection at −20°C. Samples were shipped on dry ice to the lab where they were stored at −80°C.

### (c) Fecal DNA extraction and qPCR analysis

Immediately before DNA extraction, the silica gel desiccant was removed, the feces were weighed to the nearest mg, and then homogenized with glass beads in a Mini Bead Beater (BioSpec Products) to break apart the pellets. DNA was extracted from up to 265 mg of dried, homogenized feces using the UltraClean Fecal DNA Kit (MoBio Laboratories), and the alternate lysis method followed by centrifugation for 4 min and incubation overnight at 4°C was increased from 5 minutes as recommended in the protocol. The DNA was recovered in 50 µl of elution buffer and stored at −20°C. As is common for forensic samples [28], [29], yields of DNA were generally too low to reliably quantify using a fluorometer or spectrophotometer. Because contamination is a concern for PCR of samples with very low DNA content [30], a negative control containing only extraction reagents was included in each batch of 16 extractions. qPCR amplification was performed on the samples from the captive feeding experiments (Information S1) and on a subset of 15 samples from each collection date for a total of 375 samples from free-ranging bats. Reactions were in 25 µl volumes, each containing 2 µl DNA, 1× PCR Gold buffer (Applied Biosystems), 3.5 mM MgCl_2_, 0.8 mM dNTP Blend (Applied Biosystems), 5 ng of each primer (Integrated DNA Technologies), 5 ng probe (Biosearch Technologies), 5 µg BSA (Sigma), 0.125 units of AmpliTaq Gold DNA polymerase (Applied Biosystems). The PCR amplification profile consisted of initial hot start denaturation for 10 min at 95°C, followed by 40 cycles at 95°C for 45 s, then annealing and elongation at 55.8°C for 1 min. The reactions were carried out in a Chromo4 Real-Time PCR Detection System (Bio-Rad Laboratories) and analyzed using Opticon Monitor 3 software (Bio-Rad Laboratories). Fecal samples and negative controls were run in triplicate and gene copy numbers were averaged over the triplicate runs. The qPCR results are presented as the 1) percent of sampled bats that ate CEW as assessed by the presence of the COII marker in their feces, and 2) mean numbers of CEW gene copies per mg of fecal material. Except for our greater taxonomic precision, the former is analogous to the “percent frequency,” and the latter the “percent volume” of dietary items as commonly reported after conventional fecal analyses [8], [13].

### (d) Controls for false positives and false negatives

Despite our tests to ensure the specificity of the COII primers, the diverse diet of Brazilian free-tailed bats raises concern that false positives could result if the primers inadvertently amplify analogous gene regions from other insects. As a test for false positives, qPCR products from 17 fecal samples that were scored as positive for CEW DNA were cloned, sequenced, and aligned to confirm their identity as the CEW COII gene region (Information S1).

Although studies report reliable amplification of short mt-DNA sequences of prey from the feces of predators [9], [31], compounds in feces can inhibit the PCR process and raise concerns for false negatives [32] or reduced yields of qPCR product. To test samples for false negatives due to the possible presence of PCR-inhibitors, 11 samples that were negative after qPCR, and nine samples that were positive with low to moderate gene copy numbers, were rerun after supplementing each sample with ∼200 gene copies/ul of the CEW COII gene sequence that was obtained from moth genomic DNA (Information S1).

### (e) Insect monitoring

Adult CEW populations were monitored by professional crop consultants at four sites within the Winter Garden region. These sites were on farms of cooperating growers, with two sites (Hondo and Knippa) within the estimated 100 km nightly commuting distances [22] of Brazilian free-tailed bats roosting under Seco Creek Bridge (Figure 1). At each site, a wire cone trap baited with a pheromone lure dispenser (Hercon Environmental, product #100337) to attract adult male CEW moths was placed along the perimeters of corn and cotton fields. From March 7 to October 12, 2006, moths were collected from each trap, mostly two times per week and more frequently during periods of peak moth abundance. Moth abundance was recorded as the average daily number of moths captured during each sample interval, with this average number ascribed to the mid-day of each interval.

### (f) Statistical analysis

Summary statistics are presented as mean +/− SE. Unless otherwise specified, statistical analyses were performed using JMP (JMP, Version 7. SAS Institute Inc., Cary, NC, 1989–2007). Patterns of CEW moth abundance at the four pheromone trap sites were compared using multivariate correlation analysis of the numbers of moths captured at each site on each date. Because pheromone trap dates for moths did not always correspond with sampling dates for bat feces and because there were gaps at some sites in the pheromone trapping time-line, the temporal patterns of moth abundances were fit to spline functions (Figure S2) to provide estimates of moth abundance corresponding to each fecal sampling date. Linear regression was then used to examine associations between moth abundances at each site and the percent of fecal samples positive for the CEW gene marker. As we have no apriori information on where the bats forage relative to the locations of our pheromone trap sites, the spline function estimates of moth abundance from the four sites were combined as our best indicators of the temporal patterns of CEW moth availability in the region. The estimated moth abundances from the four sites were combined in two ways: 1) the average of the estimated number of moths captured at the four sites on each sample date, and 2) the maximum of the estimated number of moths collected at any of the four sites on each sample date (Figure S2). In combining moth numbers at the four sites, we reasoned that the estimates based on the average numbers of moths captured imply that the bats forage equivalently over these sites; whereas the estimates based on maximum numbers of moths captured imply that bats forage at the locations where moths are most abundant. Linear regression then was used to examine associations between these combined estimates of moth abundance and the percent of fecal samples that were positive for the CEW gene marker, and between combined estimates of moth abundance and the natural log of the average number of CEW gene copies per mg fecal material in positive samples as estimated using qPCR. Because we were successful in all attempts to clone and sequence the CEW gene marker from fecal samples that amplified in at least one of the triplicate reactions, including the samples with the lowest estimated gene copy numbers (Information S1), we accepted as “positive” samples those that yielded the CEW gene marker in one or more qPCR reaction. All analyses were repeated under the more demanding criteria that samples had to amplify in two or in all three of the triplicate reactions to be accepted as positive.

## Results

### (a) Fecal samples from wild bats

The CEW gene marker amplified in at least one qPCR reaction from 34.4% (129 of 375) of the fecal samples collected from free-ranging bats. Fewer samples were positive for two (24.0%; 90 of 375) or for all three (17.3%; 65 of 375) of the triplicate qPCR reactions. Although differences were not significant, mean gene copy numbers were higher in samples that amplified in all triplicate reactions (Y = 26,740+/−70,577 gene copies per mg), and lower in samples that amplified in two (Y = 24,660+/−68,182), or in only one of the triplicate reactions (Y = 19,309+/−53,350). Mean gene copy numbers in qPCR-positive fecal samples ranged from a low of 10.6 to a high 7,607,284 gene copies per mg feces.

### (b) Moth abundance and bat diets

**Figure 2:**
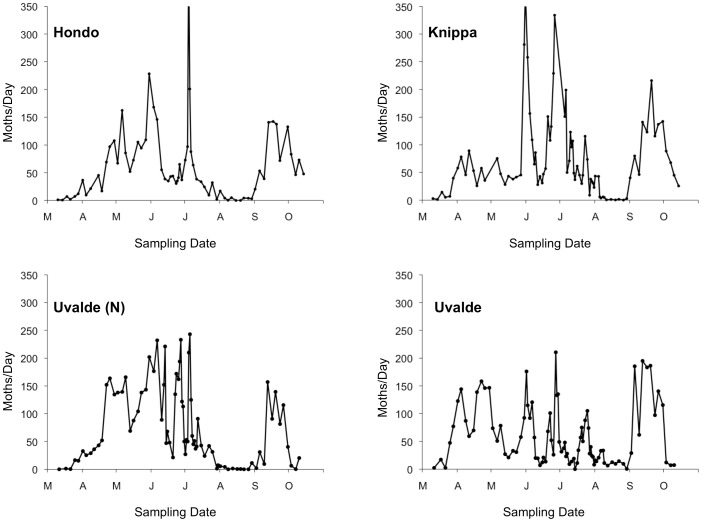
Numbers of adult male CEW moths captured in pheromone traps. Moth numbers are based on the average daily captures at four sites in Medina and Uvalde Counties, Texas, from March to October, 2006 (see text). On this and the following figures ticks and labels on the x-axis indicate the beginning of each month. The maximum number of moths captured at Hondo (N = 435) and Knippa (N = 379) exceed the scale of the figure.

The numbers of CEW moths captured in pheromone traps showed abrupt, temporal shifts in abundance (Figure 2) conforming to patterns documented by previous research in the region [18], [19], [20]. Except in March and in August, CEW moths were captured at most sites on most dates. Increases in moth abundance were pronounced at three of the four sites in late-May and early-June when migratory moths arrive in the region and infest corn, and again in late-June to early-July when moths emerge from corn and infest cotton and other crops (Figure 2). During this latter period, a maximum of 435 moths was captured in one trap in a single night. The influx of CEW moths resulting from their southward migration in September also was evident at all four sites (Figure 2). The temporal patterns of moth abundance at Knippa, Hondo, and Uvalde (N) were significantly correlated (Table 1); whereas moth abundance at Uvalde was not correlated with that at Hondo and more weakly correlated with moth abundance at the other sites (Table 1). Our pheromone trapping efforts throughout the study yielded a total of 53,914 adult CEW moths.

**Table 1:** R-squares (in bold, above diagonal) and *sample sizes (n) and p-values (below diagonal) between temporal patterns of moth abundance at each sampling site.

	Knippa	Hondo	Uvalde N	Uvalde
**Knippa**	**1**	**0.35**	**0.31**	**0.19**
**Hondo**	53, <0.0001	**1**	**0.41**	**0.02**
**Uvalde N.**	56, <0.0001	53, <0.0001	**1**	**0.18**
**Uvalde**	68, 0.0002	48, 0.3650	66, 0.0003	1

* n is the number of days in which samples were collected at each site on the same day.

Linear regressions (Table 2) show significant associations at three of the four pheromone trap sites between moth abundances and the percentages of bats with the CEW gene marker in their feces (Knippa (R^2^ = 0.398, P<0.0007); Hondo (R^2^ = 0.260, P = 0.0092); Uvalde (N) (R^2^ = 0.212, P = 0.0206)), and no association at the fourth site (Uvalde (R^2^ = 0.007, P = 0.690)). The associations between moth abundance and the percentage of bat feces that are positive for the CEW gene marker remain significant when the criteria for accepting a positive are two or three positive reactions; except at Uvalde, and at Uvalde (N) in the case of three positives (Table 2).

**Table 2:** R-squares (in bold) and F-values and p-values (in parentheses) of associations between spline function estimates of moth abundances at each sample site and the percentages of fecal samples positive for the CEW gene marker.

Criteria for positive fecal samples	Knippa	Hondo E	Uvalde N	Uvalde
All 3 positive	**0.370**	**0.171**	**0.161**	**0.022**
	(13.486, 0.0013)	(4.730, 0.0402)	(4.419, 0.467)	(0.507, 0.4836)
At least 2 positive	**0.499**	**0.331**	**0.236**	**0.015**
	(22.913, <0.0001)	(11.365, 0.0026)	(7.120, 0.0137)	(0.350, 0.560)
At least 1 positive	**0.398**	**0.260**	**0.212**	**0.007**
	(15.203, <0.0007)	(8.093, 0.0092)	(6.181, 0.0206)	(0.163, 0.690)

**Figure 3:**
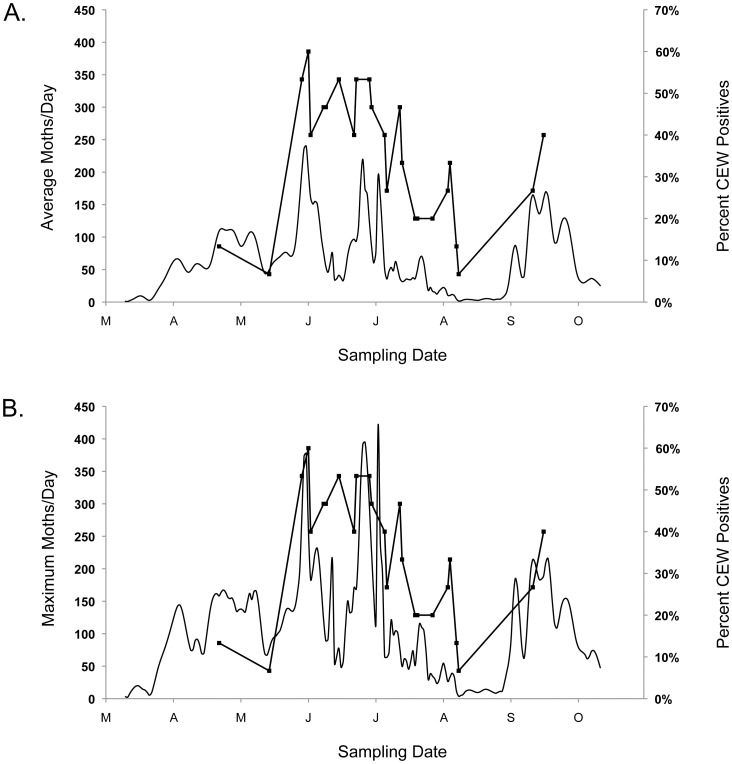
CEW moth abundance and bats positive for the CEW gene marker in their feces. Moth abundances (solid lines, left scales) are the average (A) and the maximum (B) of the estimated numbers of moths captured at any of the four pheromone trap sites. Positives (lines with squares, right scales) are the percentage of fecal samples that yielded the CEW gene marker in at least one qPCR reaction. For the data shown, the linear associations between CEW moth abundance and bats that consumed CEW are R^2^ = 0.27, P = 0.0082 and R^2^ = 0.37, P = 0.0012 for the average and maximum estimates of moth abundances, respectively (Table 3).

The spline function of moth abundance based on the average numbers of moths captured at the four sites, and the function based on the maximum numbers of moths captured at a site on each date, are both significant in explaining patterns of CEW moth consumption (Table 3). Significant associations persist for both functions at all criteria for what is accepted as a positive sample (Table 3). The spline function of moth abundance based on the maximum numbers of moths captured consistently out-performs (R^2^ = 0.37 to 0.48) the function based on capture averages (R^2^ = 0.24 to 0.35) in explaining patterns of CEW moth consumption by bats (Table 3). Overlay plots illustrate the association between the percentage of fecal samples from bats that are positive for the CEW gene marker and the seasonal patterns of moth abundance based on average and maximum numbers of moths captured at the four sites (Figure 3).

**Table 3:** R-squares (in bold) and F-values and p-values (in parentheses) of associations between spline function estimates of moth abundance at all four sample sites and the percentages of fecal samples positive for the CEW gene marker.

Criteria for positive fecal samples	Estimates of moth abundances at all 4 sites	
	Maximum	Average
All 3 positive	**0.41**	**0.24**
	(15.81, 0.0006)	(7.41, 0.0122)
At least 2 positive	**0.48**	**0.35**
	(20.85, 0.0001)	(12.18, 0.002)
At least 1 positive	**0.37**	**0.27**
	(13.76, 0.0012)	(8.38, 0.0082)

Estimates of moth abundances were combined for each sample date as the maximum number of moths captured at any site and as the average number of moths captured at all four sites.

**Figure 4:**
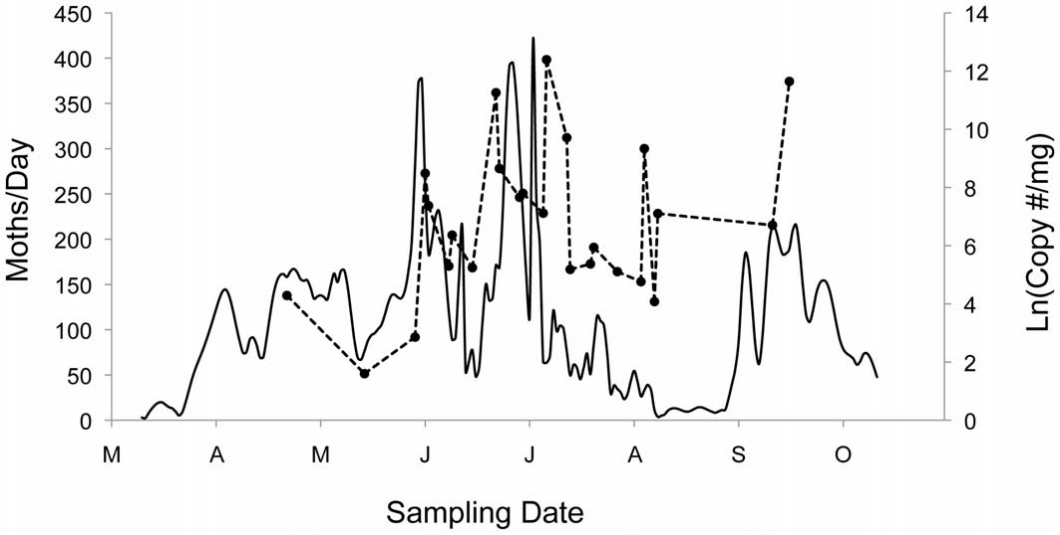
CEW moth abundance and the natural log of the average CEW gene copy number. Moth abundance (solid line, left scale) is the maximum of the estimated numbers of moths captured at any of the four pheromone trap sites. CEW gene copy numbers per mg in qPCR-positive fecal samples (dashed line, right scale) are those that yielded the CEW gene marker in at least one qPCR reaction. For the data shown, the linear association between CEW moth abundance and CEW gene copies in feces is R^2^ = 0.03, P = 0.44.

**Figure 5:**
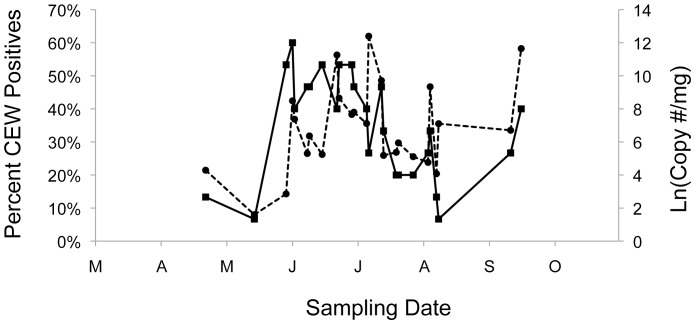
Bats positive for the CEW gene marker and CEW gene copy number. The percentage of bats sampled that were CEW positive in their feces (solid line, left scale) and the natural log of the average CEW gene copy number per mg feces (dashed line, right scale) are as in Figures 3 and 4.

Regression analyses revealed no significant associations between CEW gene copy numbers per milligram feces and the temporal patterns of moth abundance, either for moth abundances at single pheromone trap sites (results not shown), or for the spline functions of moth abundance that combine data from all trap sites (R^2^ = <0.001 to 0.026; P = 0.92 to 0.44, for maximum abundances of moths; R^2^ = 0.002 to 0.016; P = 0.84 to 0.55, for mean abundances of moths). Overlay of the seasonal patterns of moth abundance (maximum captured at sites) and average gene copy numbers/milligram feces (Figure 4) demonstrates that high gene copy numbers often occur late in the season when moths are rare. Overlay of the frequency of consumption (percent positives) versus mean gene copy numbers suggests that during early-to-mid season when more bats are feeding on CEW, lower gene copy numbers often occur in feces, whereas later in the season when proportionately fewer bats are feeding on CEW, the feces of those that do often have higher gene copy numbers (Figure 5).

## Discussion

### (a) Tracking resources

Our results demonstrate that the consumption of CEW moths by Brazilian free-tailed bats tracks the abundance of these moths. While the seasonal patterns of moth abundance differ among the pheromone trap sites, they are significantly correlated at three of the four sites, and the numbers of bats consuming CEW moths are significantly associated with moth abundances at each of these same sites. At the fourth trap site (Uvalde) moth abundance was neither correlated with those at the other sites, nor associated with the numbers of bats consuming CEW moths. Our estimates of moth abundances combining data from the four sites are indicators of the distribution of CEW moth availability over the landscape. With regard to the use of this landscape by bats, the estimates based on the maximum numbers of moths captured consistently outperform the estimates based on the average numbers of moths captured in providing the closest associations with the numbers of bats consuming CEW moths (Table 3; Figure 3). This result is expected if bats forage at the locations where moths are most abundant, and is consistent with the hypothesis that the bats track moth abundance in space as well as time.

In contrast to the significant associations observed between the numbers of bats eating CEW and estimates of CEW moth abundance, the numbers of CEW gene sequences in the feces of bats show no associations with our estimates of moth abundance (Figure 4). There are many complications in relating gene copy numbers to estimates of biomass or numbers of prey consumed [14]. These complications include differences in the DNA content of different prey species, differences in digestive efficiencies and DNA content of different body parts of prey, possible variation in DNA content related to the age or life history stage of prey items, the age of the meal that produced the feces, and inherent variation in the PCR process [14]. Our captive feeding experiments show a positive relationship between the percent mass of CEW moths in a bat's meal and the numbers of CEW gene copies in resulting feces (Information S1; Table S1; Figure S1). However, feces from meals with a similar proportional CEW content often differed by orders of magnitude in numbers of gene copies (Table S1). In addition, the relationship between gene copy content of feces and the numbers of moths eaten was affected by what else a bat ate in the same meal. This is illustrated by the separate feedings of bat # 3 (Table S1) where the consumption of five moths comprising 100% of a meal yielded over 4 million gene copies per mg feces; whereas consumption of five moths comprising 26% of a meal yielded less than 200,000 gene copies per milligram. The occurrence of such variation in qPCR estimates, even in a comparatively simple captive feeding situation, demonstrates that our attempt to calibrate qPCR gene copy numbers to CEW consumption did not provide measures of the biomass or number of prey consumed. Given that a single Brazilian free-tailed bat typically consumes many different insect taxa in a single night [8], we suspect even greater variability in gene copy numbers from field samples. Our qPCR results are consistent with the assessment by King et al. [14] that estimates of gene copies obtained in field studies are likely to provide, at best, some semiquantitative measure of predation.

It is nonetheless of interest to investigate the gene copy data in the contexts of the seasonal patterns of CEW moth abundance and the frequency of moth consumption by bats. Our results illustrate two periods, one in spring (April–early May), and the other in mid-late summer (late July–September), when moths are rare and few bats are feeding on them. However, during spring gene copy numbers are low, whereas in mid-late summer, gene copy numbers are higher. Earlier research on the insect resource base and dietary breadth of Brazilian free-tailed bats has shown greater insect diversity and broader dietary breadth in spring than in late summer [7]. Earlier work on the seasonal life history and behavior of the bats also shows that in late summer they feed for longer periods, lose body mass, and appear to be under much greater food stress than in spring [33], [34] (GFM, pers. observation). Taken together, these observations and our qPCR data suggest that, to the bats that eat them, CEW moths are a more important part of their diet in mid-late summer than in spring.

With the spike in CEW moth abundance in mid-season (late May–mid July) and again with their abrupt increase during the fall migration of moths, the numbers of bats eating CEW increases, and the high frequency of consumption is often coupled with high gene copy numbers. This suggests that in mid-season during pregnancy and lactation and in fall prior to migration CEW moths are an important part of the diet of many bats.

All earlier studies investigating the diets of Brazilian free-tailed bats and links to agronomic impacts were confined to mid-season months, focusing on the arrival of migrating moths in late May–early June and subsequent infestations of crops into summer [5], [6], [8]. This is the same period that the bats must cope with high energetic demands associated with pregnancy and lactation [35]. The returning southward migrations of insects on advancing cold fronts has been well established by entomological researchers, and in fact, some of the highest densities of moths ever recorded aloft were observed in September [36]. However, because these late-season migrants do not have the same immediate regional agronomic impact, the migrations and population dynamics of insects in autumn have been less studied than those during spring and summer. Our data are the first to implicate the late season migrations of insects on advancing cold fronts as a resource during another period that is critical for the survival of the bats; the time when they must accumulate fat reserves for their migratory flight in advance of the approaching winter.

### (b) Agronomic impacts

While our results are consistent with earlier research linking the diet of these bats on broader spatial and temporal scales to established patterns of emergence, migration, and abundance of several moth species, including adult CEW [8], [13], this earlier research was based on identification of insect fragments in feces, and did not provide taxonomic identification of moths below the order Lepidoptera. In the present study, the taxonomic precision provided by fecal DNA analysis links opportunistic feeding by bats on a single species of moth which, in this case, is one of the world's most destructive crop pests [5], [37].

Two studies have assessed the ecosystem services provided by Brazilian free-tailed bats within the Winter Garden region in Texas [5], [6]. Employing an avoided cost economic analysis, Cleveland et al. [5] estimated that the bats provide services amounting to 12% (range 2–29%) or $741,000 per year of the $4.6 to $6.4 million value of the annual cotton harvest in an eight-county region that includes Medina and Uvalde Counties (Figure 1). These services accrue from reduced damage to cotton bolls and the prevention of one or two pesticide applications per year. Notably, Cleveland et al. [5] also indicate that over 80% of the services provided by the bats accumulates in June and early July, during the period that we document peak consumption of CEW moths (Figure 3).

Federico et al. [6] used a stochastic stage-structured model coupled with simulations to examine the agronomic impact of bats on cotton production under a variety of scenarios that reflect changing practices in modern agriculture. Specifically, Federico et al. [6] addressed effects of increased adoption of transgenic (*Bacillus thuringiensis; Bt*) cotton; a major transition in cotton agriculture that by 2005 resulted in *Bt* varieties comprising ∼95% of the cotton planted in the Winter Garden region. In conventional cotton, the presence of bats was estimated to increase harvestable bolls and reduce pesticide applications for an estimated savings to cotton growers in the region of $688,000, a result similar to that of Cleveland et al. [5]. Federico et al.'s [6] models indicate that, although savings are less at an estimated $368,000, the value of having bats in the landscape persists under *Bt* cotton production, again due to reduced damage to bolls and reduced need for supplemental spraying. As an additional response variable in Federico et al.'s [6] models, in the absence of bats more CEW larvae survive to adulthood to disperse within and beyond the Winter Garden region.

Parameter values for the above analyses were taken from available literature, and both Cleveland et al. [5] and Federico et al. [6] cite data from Lee and McCracken [7], [8] that moths comprise approximately 30% of the bats diet with a two- to three-fold increase in moth abundance in their diet that begins in late May with the influx of migrating moths. Our qPCR data reflect this spike in moth consumption for CEW, with a more than two-fold increase (47% versus ∼20%) in positives for samples collected from May 30 to July 15 and samples collected outside of this period and before the influx of moths in September, with higher gene copy numbers often coincident with high incidence of consumption. Thus, our qPCR data are consistent with the assumptions and with the roles attributed to bats during the cotton production period modeled in both Cleveland et al. [5] and Federico et al. [6].

## Supporting Information

Information S1 **qPCR marker validation, captive feeding trials, and tests for false positives and false negatives.**
(DOC)

Figure S1 **Associations between the proportional mass (A) and total mass (B) of CEW in a bat's diet versus the ln average COII gene copy numbers per milligram (mg) feces.**
(TIF)

Figure S2 **Smoothing spline functions (lambda = 0.01) provide estimates of CEW abundance (moths captured/day) for each date that feces were collected from bats.** Spline functions were combined to provide estimates of CEW moth abundance (moths/day) throughout the study period for each of the four pheromone trap capture sites. Black lines show discrete data points connected with straight lines. Color lines represent functions obtained by combining spline functions estimates. (A.) Data on CEW abundance at each site related to estimates of the average numbers of CEW captured at all four sites. (B.) Data on CEW abundance at each site related to estimates of the maximum number of CEW captured at any site. Ticks and labels on the x-axis indicate the beginning of each month.
(TIF)

Table S1 **Results of captive feeding experiments showing COII gene copy numbers in feces, the numbers and mass of CEW moths eaten, and percent mass of CEW in a bat's diet.**
(DOC)

Appendix S1 **Genbank Accession numbers for cytochrome oxidase II (COII) sequences from insects confirmed to the lowest taxonomic level possible by entomologists and/or by comparing cytochrome oxidase I (COI) sequences to the Barcode of Life Database (data not shown).** Although the full 750 bp sequence of COII was obtained from a total of 69 insect taxa, identities were confirmed and sequences were submissible for only 40 taxa. For taxa with multiple individuals sequenced, unique sequences are labeled with specimen numbers.
(DOC)
