# Supplementary material for: Bats Track and Exploit Changes in Insect Pest Populations
Source: PLoS One. 2012 Aug 31;7(8):e43839. doi: 10.1371/journal.pone.0043839 (PMC3432057; doi:10.1371/journal.pone.0043839)
Supplement: Information S1 — qPCR marker validation, captive feeding trials, and tests for false positives and false negatives. (DOC) [file pone.0043839.s001.doc]

**Supplemental Information**

**qPCR Marker Validation**

## We performed PCR on the DNA of the wild-captured CEW moths and other insects to test the specificity of the primers moth COII I5Hz-F and moth COII I5Hz-R for corn earworms. The expected 158 bp COII gene region amplified from the genomic DNA of all 52 CEW moths tested. The primers did not amplify from the DNA of any other moths or insects tested, nor did they amplify from DNA extracted from wing membrane tissue of Brazilian free-tailed bats. Thus, within the limits of our tests, the primers moth COII I5Hz-F and moth COII I5Hz-R were specific to genomic DNA of CEW moths

**Captive Feeding Experiments**

We performed feeding experiments with captive bats to confirm that DNA from CEW moths could be extracted, amplified, and quantified from the feces of bats that had consumed these moths. Four big brown bats (*Eptesicus fuscus*) were trained to feed in a 5 liter glass beaker and allowed to eat 1, 3, 5, or 7 adult CEW moths for three successive days. Big brown bats were used in these experiments because they are hardier and easier to train and feed in captivity than are Brazilian free-tailed bats. After each feeding trial the bats were allowed to feed ad libitum on either mealworm or waxworm larvae, and the numbers of CEW moths eaten and the proportional mass of a bat’s diet that consisted of CEW moths was recorded. After day three, each bat was fed for two additional days on a diet consisting solely of mealworm or waxworm larvae. Bats were held individually in clean cloth bags, and feces were collected on the morning after each feeding, placed in 2 ml screw cap tubes (Corning) containing silica gel desiccant (4-10 mesh, Fisher Scientific), and stored at –80^o^ C. DNA extraction and PCR was as described. CEW were obtained from a commercial supplier (Benzon Research), and mealworms and wax worms were obtained from a local pet store.

During the first three days of the captive feeding experiment CEW moths comprised 4% to 100% of the mass of insects eaten by the four captive bats (Table S1). All of the qPCR amplifications of fecal DNA samples obtained after these twelve feedings were positive for the CEW moth COII gene marker. Average gene copy numbers/mg feces ranged from 2002 in a bat with CEW moths comprising 14% of its diet to over four million gene copies in a sample from a bat that ate only CEW moths, and varied greatly in feces from meals with similar percent masses of these moths (Table S1). There were positive associations between both the proportion (R^2^ = 0.616) and the mass (R^2^ = 0.588) of CEW moths in a bat’s diet versus the ln COII gene copy numbers in its feces (Fig S1). These associations are reduced when days that CEW were not in a bat’s diet are removed from the analysis (R^2^= 0.502 and R^2^= 0.192, respectively, for proportion and mass of CEW in diets). The COII gene marker amplified, although at much lower numbers, from the feces of the captive bats for up to two days after CEW moths were removed from their diet (Table S1).

**Testing for False Positives - Cloning of the COII Marker from Field-collected Samples**

Because AmpliTaq Gold, which was used for qPCR, is a proofreading polymerase, it does not add the 3’ adenine-overhang necessary for TA cloning. Taq DNA polymerase (Promega) (1 U) was added to 5 µL of PCR product and incubated at 72°C for 10 min to add adenine necessary for the cloning reaction. This reaction (1 µL) was used for TOPO TA cloning (Invitrogen), from which plasmids were harvested from ten to twenty *E. coli* colonies and sequenced using the BigDye v3.1 Terminator Cycle Sequencing Kit (Applied Biosystems) and the plasmid M13F primer on an ABI 3100 automated sequencer (Applied Biosystems).

Using this procedure, we cloned and recovered the COII CEW moth gene sequence from all 17 putatively positive fecal samples that were tested. These 17 samples included nine samples that were strong positives, one sample that was positive for two of the triplicate reactions, and seven samples that were scored as positives in only one triplicate reaction. The COII sequence for CEW was cloned and recovered from the weakest sample in the data set (10.6 copies/mg feces), and from strong positive samples containing up to 500,000 gene copies/mg feces.

**Testing for False Negatives – Spiking DNA Content of Putative Negative and Weak Positive Samples**

The addition of 200 copies per µl of the COII gene sequence increased the qPCR yield in all 20 samples. The increase in gene copy numbers in the 11 samples that were initially negative for the COII gene marker sample approximated what was added to the sample (average increase = 192.2 +/- 72.8 gene copies; range = 165 to 375). However, the results of subsequent qPCR’s of the nine samples that were initially positive were more variable, with disproportionately large increases in qPCR yield for most samples (average increase = 4,154 +/- 10,479 gene copies; range = 165 to 32,045).

mass of CEW in a bat’s diet.

|  |  |  |  |  |  |  |
| --- | --- | --- | --- | --- | --- | --- |
|  |  |  |  |  |  |  |
|  |  |  |  |  |  |  |
|  |  |  |  |  |  |  |
|  |  |  |  |  |  |  |
|  |  |  |  |  |  |  |
|  |  |  |  |  |  |  |
|  |  |  |  |  |  |  |
|  |  |  |  |  |  |  |
|  |  |  |  |  |  |  |
|  |  |  |  |  |  |  |
|  |  |  |  |  |  |  |
|  |  |  |  |  |  |  |
|  |  |  |  |  |  |  |
|  |  |  |  |  |  |  |
|  |  |  |  |  |  |  |
|  |  |  |  |  |  |  |
|  |  |  |  |  |  |  |
|  |  |  |  |  |  |  |
|  |  |  |  |  |  |  |
|  |  |  |  |  |  |  |

|  |  |  |  |  |  |
| --- | --- | --- | --- | --- | --- |
|  |  |  |  |  |  |
|  |  |  |  |  |  |
|  |  |  |  |  |  |
|  |  |  |  |  |  |
|  |  |  |  |  |  |
|  |  |  |  |  |  |
|  |  |  |  |  |  |
|  |  |  |  |  |  |
|  |  |  |  |  |  |
|  |  |  |  |  |  |
|  |  |  |  |  |  |
|  |  |  |  |  |  |
|  |  |  |  |  |  |
|  |  |  |  |  |  |
|  |  |  |  |  |  |
|  |  |  |  |  |  |
|  |  |  |  |  |  |
|  |  |  |  |  |  |
|  |  |  |  |  |  |
|  |  |  |  |  |  |
|  |  |  |  |  |  |
|  |  |  |  |  |  |
|  |  |  |  |  |  |
|  |  |  |  |  |  |
|  |  |  |  |  |  |
|  |  |  |  |  |  |
|  |  |  |  |  |  |
|  |  |  |  |  |  |
|  |  |  |  |  |  |
|  |  |  |  |  |  |
|  |  |  |  |  |  |
|  |  |  |  |  |  |
|  |  |  |  |  |  |
|  |  |  |  |  |  |
|  |  |  |  |  |  |
|  |  |  |  |  |  |
|  |  |  |  |  |  |
|  |  |  |  |  |  |
|  |  |  |  |  |  |
|  |  |  |  |  |  |
|  |  |  |  |  |  |
|  |  |  |  |  |  |
|  |  |  |  |  |  |
|  |  |  |  |  |  |
|  |  |  |  |  |  |
|  |  |  |  |  |  |
|  |  |  |  |  |  |
|  |  |  |  |  |  |
|  |  |  |  |  |  |
|  |  |  |  |  |  |
|  |  |  |  |  |  |
|  |  |  |  |  |  |
|  |  |  |  |  |  |
|  |  |  |  |  |  |
|  |  |  |  |  |  |
