# Supplementary figures and images for: Bats Track and Exploit Changes in Insect Pest Populations
Source: PLoS One. 2012 Aug 31;7(8):e43839. doi: 10.1371/journal.pone.0043839 (PMC3432057; doi:10.1371/journal.pone.0043839)

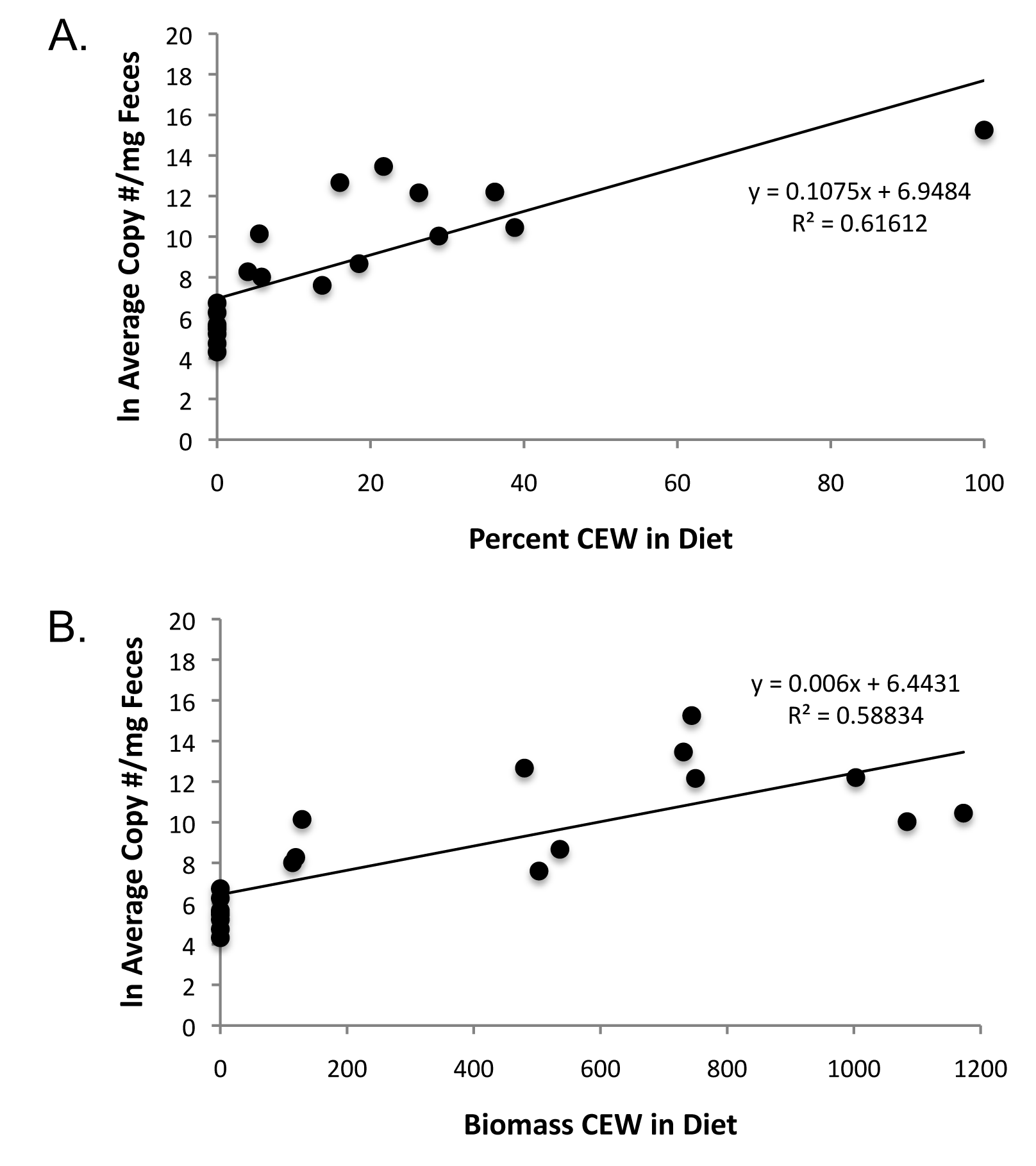

Supplement: Figure S1 — Associations between the proportional mass (A) and total mass (B) of CEW in a bat's diet versus the ln average COII gene copy numbers per milligram (mg) feces. (TIF) [file pone.0043839.s002.tif]

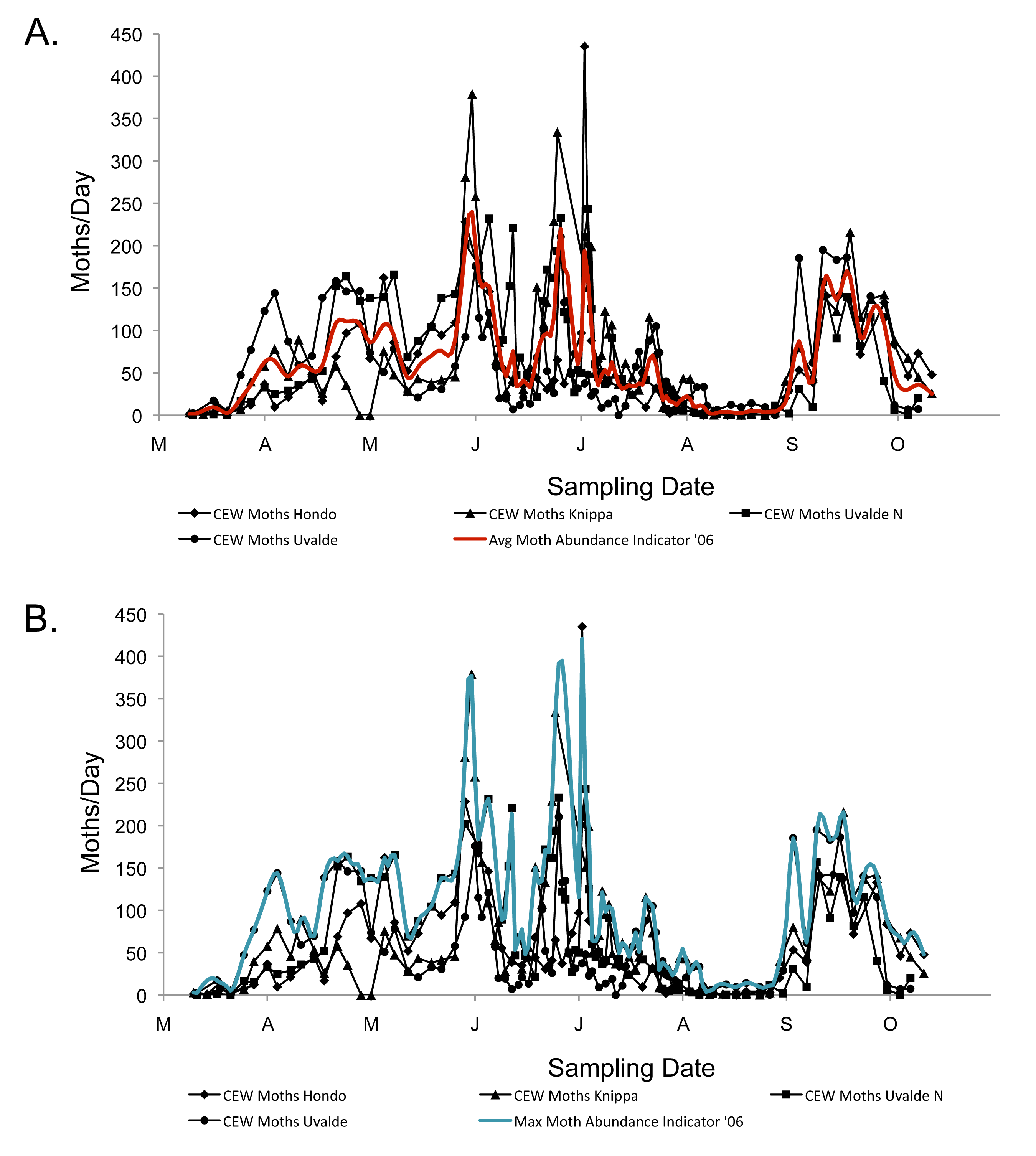

Supplement: Figure S2 — Smoothing spline functions (lambda = 0.01) provide estimates of CEW abundance (moths captured/day) for each date that feces were collected from bats. Spline functions were combined to provide estimates of CEW moth abundance (moths/day) throughout the study period for each of the four pheromone trap capture sites. Black lines show discrete data points connected with straight lines. Color lines represent functions obtained by combining spline functions estimates. (A.) Data on CEW abundance at each site related to estimates of the average numbers of CEW captured at all four sites. (B.) Data on CEW abundance at each site related to estimates of the maximum number of CEW captured at any site. Ticks and labels on the x-axis indicate the beginning of each month. (TIF) [file pone.0043839.s003.tif]
