# Supplementary material for: Bats Track and Exploit Changes in Insect Pest Populations
Source: PLoS One. 2012 Aug 31;7(8):e43839. doi: 10.1371/journal.pone.0043839 (PMC3432057; doi:10.1371/journal.pone.0043839)
Supplement: Table S1 — Results of captive feeding experiments showing COII gene copy numbers in feces, the numbers and mass of CEW moths eaten, and percent mass of CEW in a bat's diet. (DOC) [file pone.0043839.s004.doc]

**Table S1.** Results of captive feeding experiments showing COII gene copy numbers in feces, the numbers and mass of CEW moths eaten, and percent mass of CEW in a bat’s diet.

| **bat** | **day** | **# CEW** | **mass CEW (mg)** | **% CEW** | **avg copy #/mg feces** | **ln avg copy #/mg feces** |
| --- | --- | --- | --- | --- | --- | --- |
| 1 | 1 | 1 | 114 | 5.8 | 3014 | 8.011 |
|  | 2 | 1 | 129 | 5.5 | 25395 | 10.142 |
|  | 3 | 1 | 119 | 4.0 | 3910 | 8.271 |
|  | 4 | 0 | 0 | 0 | 235 | 5.460 |
|  | 5 | 0 | 0 | 0 | 286 | 5.656 |
| 2 | 1 | 3 | 536 | 18.5 | 5829 | 8.671 |
|  | 2 | 3 | 480 | 16.0 | 317388 | 12.668 |
|  | 3 | 3 | 503 | 13.7 | 2002 | 7.602 |
|  | 4 | 0 | 0 | 0 | 114 | 4.736 |
|  | 5 | 0 | 0 | 0 | 523 | 6.260 |
| 3 | 1 | 5 | 744 | 100.0 | 4210455 | 15.253 |
|  | 2 | 5 | 750 | 26.3 | 191223 | 12.161 |
|  | 3 | 5 | 731 | 21.7 | 700878 | 13.460 |
|  | 4 | 0 | 0 | 0 | 262 | 5.568 |
|  | 5 | 0 | 0 | 0 | 75 | 4.317 |
| 4 | 1 | 7 | 1173 | 38.8 | 34574 | 10.451 |
|  | 2 | 7 | 1003 | 36.2 | 199142 | 12.202 |
|  | 3 | 7 | 1084 | 28.9 | 22748 | 10.032 |
|  | 4 | 0 | 0 | 0 | 184 | 5.215 |
|  | 5 | 0 | 0 | 0 | 841 | 6.735 |
