# Supplementary material for: Bats Track and Exploit Changes in Insect Pest Populations
Source: PLoS One. 2012 Aug 31;7(8):e43839. doi: 10.1371/journal.pone.0043839 (PMC3432057; doi:10.1371/journal.pone.0043839)
Supplement: Appendix S1 — Genbank Accession numbers for cytochrome oxidase II (COII) sequences from insects confirmed to the lowest taxonomic level possible by entomologists and/or by comparing cytochrome oxidase I (COI) sequences to the Barcode of Life Database (data not shown). Although the full 750 bp sequence of COII was obtained from a total of 69 insect taxa, identities were confirmed and sequences were submissible for only 40 taxa. For taxa with multiple individuals sequenced, unique sequences are labeled with specimen numbers. (DOC) [file pone.0043839.s005.doc]

**Appendix S1.** Genbank Accession numbers for cytochrome oxidase II (COII) sequences from insects confirmed to the lowest taxonomic level possible by entomologists and/or by comparing cytochrome oxidase I (COI) sequences to the Barcode of Life Database (data not shown). Although the full 750 bp sequence of COII was obtained from a total of 69 insect taxa, identities were confirmed and sequences were submissible for only 40 taxa. For taxa with multiple individuals sequenced, unique sequences are labeled with specimen numbers.

| **order** | **family** | **genus** | **species** | **specimen** | **accession number** |
| --- | --- | --- | --- | --- | --- |
| Coleoptera | Coccinellidae | *Harmonia* | *axyridis* |  | HQ677813 |
| Coleoptera | Coccinellidae | *Hippodamia* | *convergens* |  | HQ677825 |
| Coleoptera | Scarabaeidae | *Onthophagus* | *gazella* |  | HQ677814 |
| Lepidoptera |  |  |  | 1 | HQ677800 |
| Lepidoptera |  |  |  | 2 | HQ677801 |
| Lepidoptera |  |  |  | 3 | HQ677819 |
| Lepidoptera |  |  |  | 4 | HQ677818 |
| Lepidoptera | Arctidae | *Spilosoma* | *virginica* |  | HQ677812 |
| Lepidoptera | Arctidae | *Virbia* | *aurantiaca* |  | HQ677799 |
| Lepidoptera | Crambidae | *Diacme* | *elealis* |  | HQ677803 |
| Lepidoptera | Crambidae | *Elophila* | *obliteralis* |  | HQ677804 |
| Lepidoptera | Crambidae | *Palpita* | *quadristigmalis* |  | HQ677823 |
| Lepidoptera | Crambidae | *Parapediasia* | *teterrellus* |  | HQ677807 |
| Lepidoptera | Erebidae | *Caenurgia* | *chloropha* |  | HQ677809 |
| Lepidoptera | Noctuidae |  |  | 1 | HQ677797 |
| Lepidoptera | Noctuidae | *Agrotis* | *ipsilon* |  | HQ677794 |
| Lepidoptera | Noctuidae | *Agrotis* | *malefida* |  | HQ677822 |
| Lepidoptera | Noctuidae | *Bulia* | *deducta* |  | HQ677821 |
| Lepidoptera | Noctuidae | *Cobubatha* |  |  | HQ677805 |
| Lepidoptera | Noctuidae | *Cucullia* |  |  | HQ677810 |
| Lepidoptera | Noctuidae | *Elaphria* |  |  | HQ677802 |
| Lepidoptera | Noctuidae | *Eubolina* | *impartialis* |  | HQ677817 |
| Lepidoptera | Noctuidae | *Helicoverpa* | *armigera* |  | HQ677778 |
| Lepidoptera | Noctuidae | *Helicoverpa* | *zea* | 1 | HQ677771 |
| Lepidoptera | Noctuidae | *Helicoverpa* | *zea* | 2 | HQ677772 |
| Lepidoptera | Noctuidae | *Helicoverpa* | *zea* | 3 | HQ677773 |
| Lepidoptera | Noctuidae | *Helicoverpa* | *zea* | 4 | HQ677774 |
| Lepidoptera | Noctuidae | *Helicoverpa* | *zea* | 5 | HQ677775 |
| Lepidoptera | Noctuidae | *Helicoverpa* | *zea* | 6 | HQ677776 |
| Lepidoptera | Noctuidae | *Helicoverpa* | *zea* | 7 | HQ677777 |
| Lepidoptera | Noctuidae | *Heliothis* | *subflexa* |  | HQ677779 |
| Lepidoptera | Noctuidae | *Heliothis* | *virescens* | 1 | HQ677780 |
| Lepidoptera | Noctuidae | *Heliothis* | *virescens* | 2 | HQ677781 |
| Lepidoptera | Noctuidae | *Heliothis* | *virescens* | 3 | HQ677782 |
| Lepidoptera | Noctuidae | *Heliothis* | *virescens* | 4 | HQ677783 |
| Lepidoptera | Noctuidae | *Leucania* | *incognita* |  | HQ677795 |
| Lepidoptera | Noctuidae | *Melipotis* |  |  | HQ677820 |
| Lepidoptera | Noctuidae | *Melipotis* | *jucunda* |  | HQ677798 |
| Lepidoptera | Noctuidae | *Mythimna* | *unipuncta* |  | HQ677824 |
| Lepidoptera | Noctuidae | *Spodoptera* | *frugiperda* | 1 | HQ677788 |
| Lepidoptera | Noctuidae | *Spodoptera* | *frugiperda* | 2 | HQ677789 |
| Lepidoptera | Noctuidae | *Spodoptera* | *frugiperda* | 3 | HQ677790 |
| Lepidoptera | Noctuidae | *Spodoptera* | *frugiperda* | 4 | HQ677791 |
| Lepidoptera | Noctuidae | *Spodoptera* | *frugiperda* | 5 | HQ677792 |
| Lepidoptera | Noctuidae | *Spodoptera* | *exigua* | 1 | HQ677785 |
| Lepidoptera | Noctuidae | *Spodoptera* | *exigua* | 2 | HQ677786 |
| Lepidoptera | Noctuidae | *Spodoptera* | *exigua* | 3 | HQ677787 |
| Lepidoptera | Noctuidae | *Tarachidia* | *phecolisca* |  | HQ677808 |
| Lepidoptera | Noctuidae | *Trichoplusia* | *ni* | 1 | HQ677815 |
| Lepidoptera | Noctuidae | *Trichoplusia* | *ni* | 2 | HQ677816 |
| Lepidoptera | Pyralidae | *Acrobasis* | *nuxvorella* |  | HQ677784 |
| Lepidoptera | Pyralidae | *Lygropia* |  |  | HQ677811 |
| Lepidoptera | Saturniidae |  |  |  | HQ677793 |
| Lepidoptera | Sphingidae | *Linternia* | *istar* |  | HQ677796 |
| Lepidoptera | Xyloryctidae |  |  |  | HQ677806 |
